# Nanotechnology for Drought Mitigation and Water Conservation: Opportunities and Limitations

**DOI:** 10.3390/nano16090523

**Published:** 2026-04-26

**Authors:** Hassan El-Ramady, Daniella Sári, Tamer Elsakhawy, Neama Abdalla, Howaida I. Abd-Alla, József Prokisch

**Affiliations:** 1 Soil and Water Department, Faculty of Agriculture, Kafrelsheikh University, Kafr El-Sheikh 33516, Egypt; hassan.elramady@agr.kfs.edu.eg; 2 Nanofood Laboratory, Department of Animal Husbandry, Faculty of Agricultural and Food Sciences and Environmental Management, Institute of Animal Science, Biotechnology and Nature Conservation, University of Debrecen, 138 Boszormenyi Street, 4032 Debrecen, Hungary; saridaniella91@gmail.com; 3 Microbiology Department, Soil, Water and Environment Research Institute, Sakha Agricultural Research Station, Agriculture Research Center, Kafr El-Sheikh 33717, Egypt; drelsakhawy@arc.sci.eg; 4 Plant Biotechnology Department, Biotechnology Research Institute, National Research Centre, 33 El Buhouth St., Dokki, Giza 12622, Egypt; 5 Chemistry of Natural Compounds Department, Pharmaceutical and Drug Industries Research Institute, National Research Centre, 33 El Buhouth St., Dokki, Giza 12622, Egypt; howaida.nrc@gmail.com

**Keywords:** abiotic stress, biotic stress, nano-mitigation, nano-biotechnology, water scarcity

## Abstract

Water scarcity is becoming an increasingly critical global challenge, driven by climate change, rapid population growth, pollution, and unsustainable water use. Drought further intensifies this crisis by reducing water availability across agricultural, environmental, and socio-economic systems. In this context, nanotechnology has emerged as a promising tool for improving water management and enhancing drought resilience. This review examines the role of nanotechnology in drought mitigation and water conservation through multiple pathways, including the enhancement of plant drought tolerance, improvement in soil water retention, the development of smart irrigation and nano-sensing systems, and the expansion of water resources through purification, desalination, and wastewater reuse. In addition, the broader drought–water nexus is discussed to position nano-enabled approaches within existing water management strategies. While numerous studies report improvements in water-use efficiency, stress tolerance, and treatment performance under controlled conditions, significant limitations remain. These include concerns related to environmental safety, nanotoxicity, scalability, cost, and the gap between laboratory findings and field-level applications. Overall, nanotechnology should be considered a complementary approach rather than a stand-alone solution for addressing water scarcity under drought conditions. Future research should focus on long-term environmental impacts, techno-economic feasibility, and large-scale field validation to support the safe and effective integration of nanotechnology into sustainable water management systems.

## 1. Introduction

The global water crisis has become one of the most pressing challenges of the twenty-first century, driven by climate change, increasing pollution, inefficient water use, and rapid population growth. This challenge is particularly severe in arid and semi-arid regions, where water resources are already limited and highly vulnerable to climatic variability. This crisis refers to the significant decline in the availability, accessibility, and quality of freshwater resources, leading to serious consequences for human health, economic development, and environmental sustainability [1]. Climate change accelerates drought intensity by increasing temperature, enhancing evapotranspiration, and altering precipitation patterns, thereby directly amplifying water scarcity [2]. Water consumption varies widely across regions, with a global average of approximately 4000–5000 m^3^ per person per year, compared to about 720 m^3^ per person per year in the Middle East and less than 200 m^3^ per person per year in severely water-scarce countries such as Jordan and Yemen [3]. These combined effects demonstrate that drought is not only a climatic phenomenon but also a key driver of the global water crisis, linking environmental stress with agricultural and socio-economic vulnerability. Projections indicate that global water demand may increase by 20–30% by 2050 relative to current levels [4]. In addition to water scarcity, declining water quality further complicates access to safe and clean water for agricultural, domestic, and industrial uses [5]. Consequently, the global water crisis is increasingly recognized as a critical environmental and societal issue [6]. Among the key drivers of this crisis, drought represents one of the most critical and recurring stressors affecting water availability worldwide.

Drought plays a significant role in intensifying this crisis, particularly under changing climatic conditions. As one of the most severe natural hazards, drought is associated with increased evaporation, reduced precipitation, and greater pressure on already limited water resources [2]. Its impacts are especially evident in agriculture, where water shortages directly affect crop productivity and food security, particularly in regions that rely heavily on irrigation [7]. Addressing drought requires a combination of mitigation strategies aimed at reducing its adverse effects on water resources, ecosystems, and human communities [8]. These strategies include nature-based solutions, integrated water resource management, technological innovations, and agricultural adaptation to increasingly frequent and severe drought events [8,9,10,11]. However, despite their effectiveness, these strategies are often limited by high implementation costs, technological complexity, and limited scalability, particularly in developing and water-scarce regions.

Nature-based solutions involve approaches such as ecosystem restoration, green infrastructure, and enhancement of soil ecosystem services [12,13,14]. Technological innovations, on the other hand, include tools such as artificial intelligence and machine learning for drought forecasting, remote sensing technologies, and probabilistic modeling techniques [15,16,17]. In parallel, agricultural adaptation focuses on the development of drought-resilient crops and improved management practices to sustain productivity under water-limited conditions [18]. Consequently, there is an increasing need for innovative and efficient approaches that can complement existing strategies and enhance water-use efficiency under drought conditions.

In recent years, nanotechnology has emerged as a promising approach for addressing drought and improving water management. Several studies have demonstrated that nanotechnology can contribute to drought mitigation through mechanisms such as nanoparticle-mediated enhancement of plant stress tolerance and the development of nano-enabled irrigation systems [19,20]. These approaches can support multiple aspects of agricultural systems, including soil health improvement, precision agriculture, and the enhancement of plant physiological responses under stress conditions [21,22,23]. In this context, nano-based strategies can complement existing approaches, including nature-based solutions and advanced water management techniques, to improve water-use efficiency and reduce drought impacts [9,20,24]. In addition, farmer behavior and decision-making play an important role in the successful adoption of drought adaptation strategies, contributing to sustainable agricultural development [25]. Nevertheless, the application of nanotechnology remains associated with several challenges, including concerns related to environmental safety, nanotoxicity, cost, and the limited validation of results under field conditions.

Therefore, this review aims to critically examine the global water crisis and its relationship with drought, with particular emphasis on the role of nanotechnology in water conservation. The review discusses the impacts of drought on agriculture, ecosystems, and society, and examines both nature-based and nano-enabled strategies for drought mitigation and adaptation. It also highlights current limitations and identifies future research directions for the effective and sustainable application of nanotechnology in water management under drought conditions.

## 2. Global Water Crisis: Causes and Drivers

**Figure 1:**
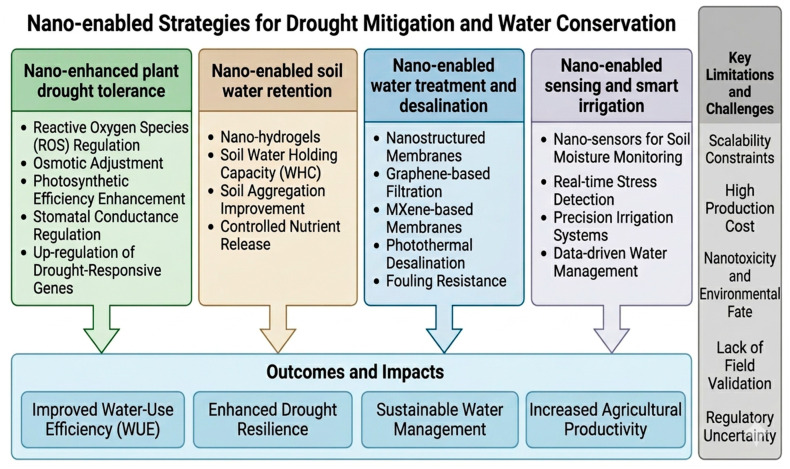
Conceptual framework illustrating the major drivers of the global water crisis. The interconnected roles of climate change, population growth and urbanization, agricultural expansion, industrialization and economic development, water pollution, and governance and socio-economic factors are presented. These drivers collectively intensify water scarcity by increasing water demand, degrading water quality, and placing additional pressure on surface and groundwater resources. Interactions among these factors, particularly the feedback between climate change and water scarcity, further amplify the complexity and severity of the global water crisis.

The global water crisis is a multi-dimensional problem driven by interconnected climatic, environmental, socio-economic, technological, and governance-related factors. The major causes, drivers, and potential solutions are summarized in Figure 1. Among these drivers, climate change is one of the most consistently documented contributors to water scarcity. It alters precipitation patterns, increases the frequency and intensity of droughts and floods, and enhances evaporation rates, thereby reducing water availability [26]. In addition, climate-induced changes in recharge patterns increase reliance on groundwater resources, leading to aquifer depletion and intensified groundwater stress [27]. Extreme climatic variability further exacerbates water scarcity and contributes to the emergence of water conflict hotspots [28]. Population growth is another major driver of the global water crisis, significantly increasing water demand across domestic, agricultural, and industrial sectors. Rapid urbanization places substantial pressure on municipal water systems, often resulting in shortages and unequal distribution. It is projected that the number of people living in urban water-scarce conditions may nearly double by 2050, while high population densities in megacities accelerate groundwater depletion and water stress [29].

Agriculture remains the largest consumer of freshwater globally, accounting for approximately 70% of total freshwater withdrawals. However, inefficient irrigation practices can result in the loss of more than 50% of water in some regions. This overuse contributes significantly to groundwater depletion and intensifies blue-water scarcity across many river basins [30]. In parallel, industrialization and economic development further increase water demand and pollution. Industrial activities, particularly those associated with thermal power generation, require substantial water for cooling processes, thereby increasing pressure on water resources. Moreover, industrial discharge contributes to surface and groundwater contamination, reducing the availability of usable freshwater [31,32]. Water pollution is a critical factor contributing to the decline in water quality and the reduction in usable freshwater supplies. Pollution originating from agricultural, industrial, and domestic sources restricts water availability, while chemical contaminants such as salinity, nutrients, and toxic compounds further exacerbate water scarcity. In addition, emerging pollutants and inadequate wastewater management practices increase risks to water security [33].

Groundwater depletion represents another major dimension of the global water crisis. Groundwater provides approximately 50% of drinking water worldwide and supports significant portions of industrial and agricultural activities. However, excessive extraction driven by population growth, agricultural demand, and climate-related pressures has led to widespread depletion of aquifers [34]. Governance-related challenges also play a crucial role in intensifying the global water crisis. Poor water management practices, inadequate infrastructure, and ineffective or inequitable policies often result in inefficient water allocation and distribution failures. In addition, transboundary water conflicts and weak institutional cooperation further increase risks associated with water scarcity. These governance deficiencies can significantly amplify the likelihood of water-related conflicts [35,36]. Socio-economic inequality and cultural factors further contribute to water scarcity by increasing vulnerability to water-related shocks. Poverty limits access to reliable water resources, while cultural and institutional practices can influence water-use behavior in ways that are not always sustainable. As a result, marginalized communities are disproportionately affected by water scarcity and its associated impacts [37].

Recent studies have examined the global water crisis from various perspectives, highlighting its complexity and multi-sectorial nature. These include investigations into the role of bioenergy carbon capture and storage in water resource security [38], potable water safety challenges [39], fluoride contamination as a public health concern in Africa [40], the potential of nanofertilizers as a strategy for sustainable agriculture under water stress [41], the impacts of microplastic and nanoplastic pollution in marine ecosystems [42], and the role of microplastic pollution as an emerging environmental threat to water security in the Global South [33].

## 3. Drought and Global Water Crisis

This section highlights drought and its contribution to the global water crisis, how drought drives the global water crisis, the main mechanisms or pathways of such impacts and the major adaptation or/and mitigation strategies. Drought and the global water crisis are deeply interconnected, mutually reinforcing challenges driven by climate change, unsustainable water use, and socio-economic pressures. Drought can also emerge as both a direct climatic driver of water scarcity and a societally amplified stressor that interacts with land-use change and growing water demand. Droughts can reduce water availability across surface and groundwater systems, intensify competition between users, and accelerate the transition from physical scarcity to full-scale water crises [43,44]. The main drought drivers under global water scarcity may include climate change and hydrological, ecological, socio-economic and human drivers. Climate change might induce drought, increasing long-term water stress. Rising temperatures intensify atmospheric evaporative demand and increase hydrological and agricultural drought severity across multiple regions. Climate change alters precipitation patterns and increases extreme events (both floods and droughts), and future projections show heightened drought frequency, including rapid-onset flash droughts across 74% of regions [45,46,47].

The global water crisis could be intensified by five major interacting drivers, including population and economic growth, climate change, agricultural expansion, pollution, and geopolitical tensions. These drivers collectively worsen scarcity, degrade quality, and destabilize water governance. There are seven types of adaptation and mitigation strategies, including (1) integrated drought management by multi-index monitoring, early warnings, and risk governance frameworks [1,7]; (2) agricultural adaptation by applying drip irrigation, agronomic adjustments, drought-tolerant crops, soil moisture conservation, and climate-smart agriculture (CSA) adoption [48]; (3) nature-based solutions and ecosystem resilience through vegetation restoration and green infrastructure for urban drought resilience [49]; (4) groundwater management by monitoring, AI-based forecasting, policies to reduce over-extraction [50]; (5) water conservation and demand management through behavioral responses to scarcity and societal adoption of water conservation narratives [51]; (6) policy and institutional measures by drought policies, common agricultural policy (CAP) reforms in the EU, and improving governance structures [52]; and (7) technological innovations using machine learning (ML) and deep learning (DL) forecasting, remote sensing, and cyber-infrastructure for drought data [53,54].

**Figure 2:**
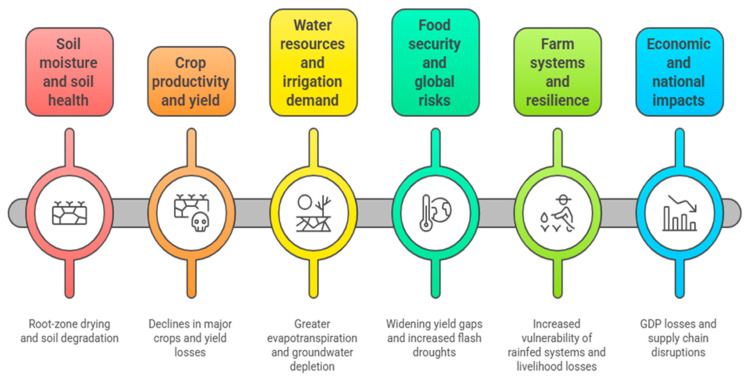
Key impacts of drought on soil systems, crop productivity, water resources, and socio-economic stability. The cascading effects of drought include degradation of soil moisture and soil health, reductions in crop yield, increased irrigation demand and groundwater depletion, and broader consequences for food security, farm resilience, and national economies. These interconnected impacts highlight the multi-scale influence of drought on agricultural systems and global sustainability.

It is well known that drought emerges as one of the most damaging climate-related hazards for agriculture, affecting food security, crop yields, agricultural economics, irrigation demand, and farming systems (Figure 2). Extreme drought can cause severe reductions in crop yields by 20–50% [55,56], reduced soil moisture and health [57,58], reduced soil water availability, soil degradation and reduced microbial activity [59], increased evapotranspiration, groundwater depletion, higher irrigation demand and water-use efficiency changes [60,61], increased vulnerability of rainfed systems, crop pattern shifts [62,63], reduced crop productivity, widened yield gaps, increased frequency of flash droughts [64,65,66], and losses to agricultural GDP, employment, and household income [67].

**Figure 3:**
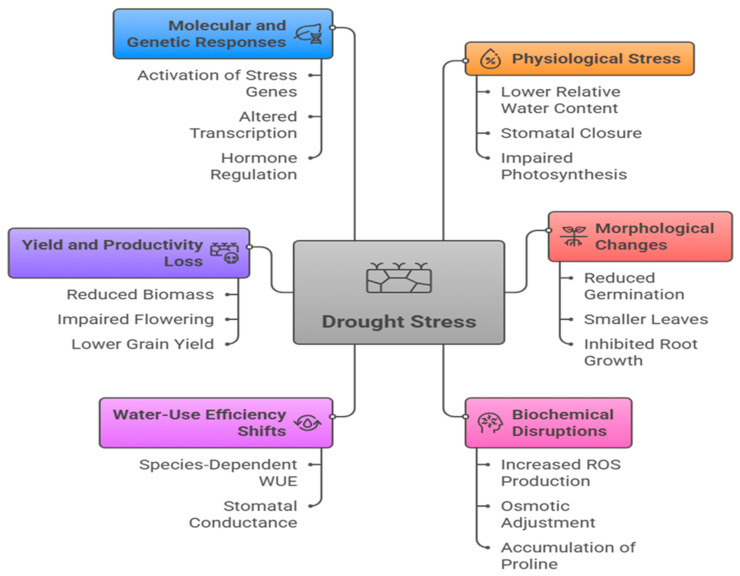
Physiological, morphological, biochemical and molecular responses of plants to drought stress. The multi-level effects of drought, including physiological stress (e.g., reduced water status and photosynthesis), morphological changes (e.g., inhibited growth and leaf development), biochemical disruptions (e.g., oxidative stress and osmotic adjustment), and shifts in water-use efficiency, yield and productivity losses, as well as molecular and genetic responses. These interconnected processes collectively determine plant performance and adaptation under drought conditions.

The impact of drought on the agricultural sector is an important issue that may lead to a global water crisis. This is confirmed by several recent publications, which focused on different themes such as the monitoring of drought using remote sensing [68], real-world agricultural drought records and machine learning [69], multi-timescale drought indices [70], atmospheric dryness and flash drought [71], explainable machine learning algorithms [61], a self-organizing agricultural drought index [72], and a multi-modal ensemble approach for forecasting agricultural droughts [73]. The impact of drought on cultivated plants is considered one of the most important issues in the agricultural sector. Drought can reduce plant growth and productivity through interconnected physiological, morphological, biochemical, molecular, and metabolic disruptions (Figure 3). These effects arise from water deficit, osmotic imbalance, oxidative stress, and altered hormonal signaling. Morphological, physiological, molecular and hormonal impacts in plants under drought were reported by several investigators [74,75,76,77,78,79,80,81,82].

**Figure 4:**
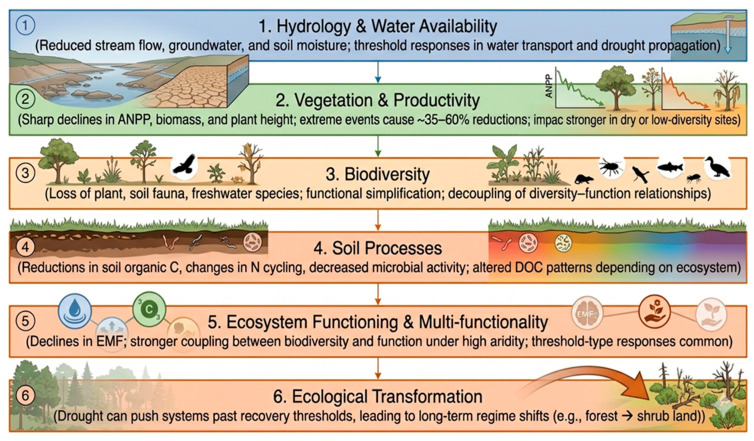
Cascading impacts of drought on ecosystem structure and functioning across multiple scales. Impacts of drought on hydrology and water availability can lead to declines in vegetation productivity and biodiversity, alterations in soil processes, and reductions in ecosystem functioning and multi-functionality. These interconnected responses may ultimately drive ecological transformation, pushing ecosystems beyond recovery thresholds and resulting in long-term regime shifts.

Drought emerges as a major ecological disturbance that affects ecosystem functions, biodiversity, species interactions, soil processes, and long-term ecosystem resilience. The impacts of drought are widespread across grasslands, forests, freshwater systems, lakes, and soil-based ecosystems (Figure 4). Biodiversity loss and species shifts might lead to the elimination of drought-intolerant species, a reduction in above- and below-ground biodiversity, an increase in plankton dissimilarity, alterations in species composition, and increased fish mortality in heat-amplified droughts [83,84]. Reducing vegetation and productivity by 21–35% in grasslands/shrub lands due to extreme drought reduces productivity, and concurrent droughts drive significant global net primary production loss. Several studies have been published about the impacts of drought on different ecosystems [85,86,87,88,89,90,91,92,93,94,95,96,97]. Drought affects society through intertwined social, economic, and health pathways, often reinforcing existing vulnerabilities on both global and national levels. The impacts of drought on public health may include increased infant mortality, malnutrition, cholera risk, poor sanitation, women’s and children’s health deterioration, mental health decline, reduced water quality, reduced wellbeing, and water-, food-, and vector-borne diseases [98,99,100,101,102]. Drought consistently undermines agricultural output, which increases food prices and reduces incomes for farmers and farm-dependent communities. Economic consequences appear on the global level depending on the country’s development level through impacts on water, energy, and industrial sectors, with variable effects on gross domestic product as well as more stress on social stability and inequality [103,104,105,106].

## 4. Nature-Based Drought Solutions

**Figure 5:**
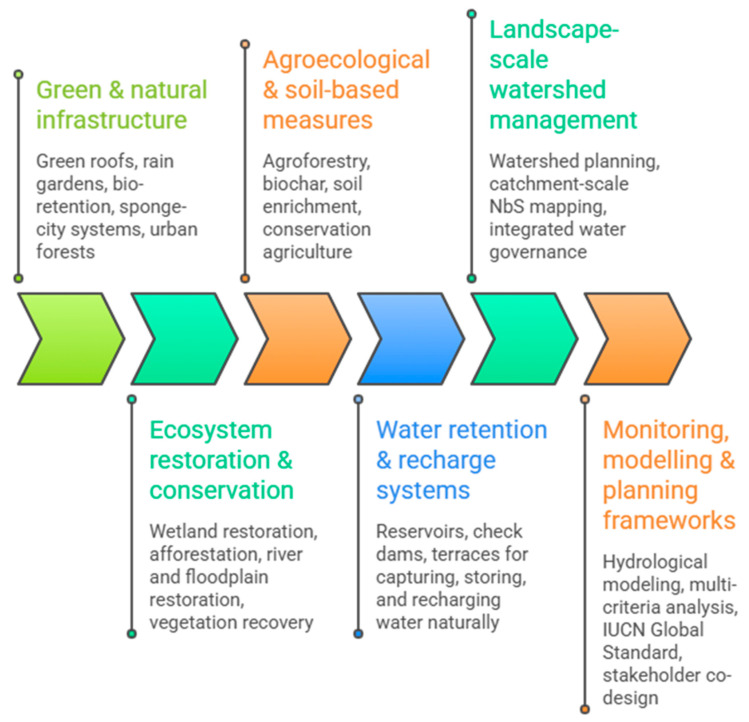
Nature-based and ecosystem-based solutions for drought mitigation and sustainable water management. A range of integrated approaches is presented, including green and natural infrastructure, ecosystem restoration and conservation, agro-ecological and soil-based practices, water retention and recharge systems, landscape-scale watershed management, and monitoring and planning frameworks. Together, these strategies enhance water retention, improve ecosystem resilience, and support adaptive, sustainable responses to drought across multiple spatial scales.

What do nature-based solutions mean, and how can they mitigate drought? What are the types of nature-based drought solutions, and what can they achieve? The concept of “nature-based solutions” was developed during the United Nations Framework Convention on Climate Change (UNFCCC) negotiations in 2009 and was introduced in the 2013–2016 International Union for Conservation of Nature (IUCN) Global Program. Nature-based solutions (NbSs) can be defined as actions that protect, restore, or sustainably manage ecosystems to address societal challenges such as biodiversity loss, climate change, urban resilience and water security [107]. NbSs are considered effective for climate adaptation through reducing climate-related risks (erosion, drought, floods and heat) across multiple ecosystems [108]. Nature-based drought solutions broadly aim to restore ecosystems, enhance water retention, improve landscape hydrology, strengthen resilience, and provide co-benefits for biodiversity and communities [109,110]. Suggested nature-based drought solutions are presented in Figure 5. For the restoration of ecosystems (e.g., grassland, forest, and peat land), nature-based drought solutions are useful for improving biodiversity, soil stability, ecosystem resilience, and carbon sequestration [111,112]. Water-focused nature-based solutions (e.g., wetlands, recharge channels, rainwater harvesting, and watershed restoration) can enhance water availability, regulate runoff, improve water quality, and increase climate resilience [113,114,115,116,117,118]. Nature-based solutions for agricultural land-use harness ecological processes (through soil formation, biodiversity, water regulation and nutrient cycling) to improve resilience, restore productivity, and address climate, water, and soil challenges. This can increase soil health and water quality, supporting sustainable livelihoods [119,120].

## 5. Drought–Water–Nanotechnology Nexus

In general, the drought–water and nanomaterials nexus refers to how nanotechnology intervenes simultaneously in drought mitigation and water conservation, linking plant-level drought tolerance, soil water retention, and nano-enabled water treatment. Nanomaterials (NMs) have the ability to reduce agricultural water demand, increase supply through purification/reuse, and enhance resource-use efficiency. Nanomaterials can also act on this nexus through three interconnected domains: (i) drought mitigation in crops, (ii) soil water retention and water delivery systems, and (iii) water treatment, reuse, and desalination. What about the nexus mapping, and how nanomaterials can interlink drought and water systems? This could be achieved by NMs enhancing plant drought tolerance by improving NMs to water status, antioxidant defense, photosynthesis, and osmotic balance across diverse crops (e.g., nano-enabled osmotic adjustment, NPs of metals). Applying NMs in soil water retention and conservation can increase the water availability to crops and reduce irrigation demand (e.g., cellulose nano-fibers, nano-enabled drip irrigation, carbon nano-fibers, etc.). On the other hand, nanotechnology can be applied for water treatment, reuse, and desalination [121,122]. NMs expand water supply through purification, desalination, and recycling using nano-enabled membranes, and microbial–nanotechnology systems enable bioremediation and nano-assisted pollutant removal.

## 6. Nano-Mitigation for Drought

**Figure 6:**
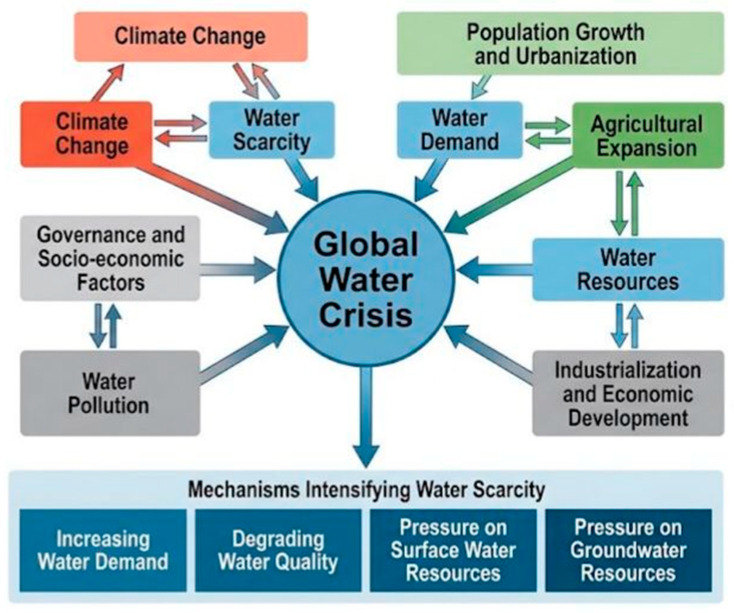
Nano-enabled strategies for drought mitigation and water conservation.

Nanotechnology has emerged as a promising approach for mitigating drought stress and improving water-use efficiency through multiple interconnected physiological, biochemical, and technological pathways. These include the enhancement of plant drought tolerance, improvement in soil water retention, development of smart irrigation systems, and advancement of water treatment technologies. However, despite these advances, most reported benefits remain derived from controlled laboratory conditions and are often influenced by nanoparticle type, concentration, plant species, and environmental context. How can nanotechnology mitigate drought? Different types of nanoparticles (NPs), nano-structured hydrogels and nano-enabled materials can improve crop performance under drought stress through multiple interconnected mechanisms [123]. Nanotechnology offers multiple complementary pathways to mitigate drought stress in plants and soils and can mitigate such stress primarily by improving soil water retention, enhancing plant stress tolerance by improving plant water-use efficiency and physiological resilience, delivering nutrients more efficiently under water deficit, and supporting soil–microbe–plant interactions that strengthen drought tolerance [20,124]. These nano-solutions operate through water retention, controlled nutrient delivery, plant physiological enhancement, and smart irrigation/monitoring (Table 1). Despite these promising outcomes, it is important to note that the majority of reported benefits are derived from controlled laboratory and greenhouse experiments, often conducted under simplified and highly regulated conditions. Such environments do not fully capture the complexity and variability of field conditions, including soil heterogeneity, climatic fluctuations, and biotic interactions. Consequently, the reproducibility and scalability of these results under real agricultural systems remain uncertain (Figure 6).

### 6.1. Nano-Enhanced Plant Drought Tolerance

Nanomaterials (NMs) enhance plant tolerance to drought stress through multiple interconnected physiological, biochemical, and molecular mechanisms. These mechanisms collectively improve plant water status, stress resilience, and productivity under water-deficit conditions, while remaining highly dependent on nanoparticle properties and application conditions. Applied NMs can mitigate drought stress through the following key mechanisms: improvement in plant water relations by enhancing plant water status through increasing water-use efficiency (WUE), relative water content (RWC), and root hydraulic conductance, thereby improving water uptake and retention under drought conditions [123]; the regulation of oxidative stress and reactive oxygen species (ROS) and enhancing antioxidant defense systems, leading to decreased lipid peroxidation (MDA) and hydrogen peroxide accumulation [136]—for example, carbon quantum dots (CQDs) have been shown to activate antioxidant enzymes and improve redox homeostasis under drought stress [137]; the enhancement of photosynthesis and gas exchange or the support of photosynthetic activity by restoring chlorophyll content, improving stomatal conductance, and stabilizing electron transport processes, thereby maintaining carbon assimilation under drought conditions [138]; osmotic adjustment via osmolyte accumulation and using NPs to promote the accumulation of osmolytes such as proline and soluble sugars, helping to maintain cell turgor and osmotic balance under water-deficit conditions [137]; the up-regulation of drought-responsive genes and hormonal signaling, where NPs activate stress-responsive genes and influence hormonal pathways, particularly abscisic acid (ABA)-mediated signaling, thereby strengthening intrinsic drought-response mechanisms and regulating stomatal behavior [139,140]; the enhancement of nutrient uptake and improvement in the acquisition of essential nutrients by NPs, including nitrogen, phosphorus, and micronutrients, which are often limited under drought conditions, thereby supporting plant growth and metabolic activity [141]; and the modulation of the rhizosphere microbiome, influencing rhizospheric microbial communities via NPs and improving nutrient availability and water uptake efficiency through enhanced plant–microbe interactions [142]. Commonly studied NMs include ZnO, SiO_2_, TiO_2_, and carbon-based nanomaterials, which have demonstrated varying effectiveness in improving drought tolerance [143]. However, these responses are strongly dose-dependent and species-specific, with experimental studies highlighting optimal concentration thresholds and potential toxicity at higher doses [137]. Therefore, standardized dose–response relationships and cross-species validation remain critical research priorities. While these mechanisms are well-documented, most supporting evidence originates from short-term experiments under controlled conditions. Field-based validation across different crops, soil types, and climatic zones remains limited. This gap raises important questions regarding the long-term stability, optimal dosing, and consistency of nanoparticle performance under practical agricultural conditions.

### 6.2. Nano-Enabled Soil and Water Retention

Nano-enabled hydrogels and soil conditioners represent an important pathway for improving soil water retention and mitigating drought stress. Superabsorbent hydrogels can significantly enhance soil moisture availability by reducing evaporation losses and maintaining water supply within the rhizosphere [144]. In addition to improving water retention, these materials influence soil microbial activity and nutrient cycling by modifying soil physical and chemical properties. Hydrogels can also function as slow-release carriers for nutrients and agrochemicals, thereby improving nutrient-use efficiency and reducing leaching losses [140]. Despite these advantages, most evidence remains limited to laboratory and small-scale studies. Field-scale validation is still scarce, and long-term impacts related to biodegradability, soil structure, and ecological interactions remain insufficiently understood. These uncertainties highlight the need for multi-season field trials and lifecycle assessments. However, the reported improvements in soil water retention are largely based on laboratory-scale studies or controlled pot experiments. The long-term behavior of nano-enabled hydrogels under repeated wetting–drying cycles, their interactions with native soil microbiota and their persistence under field conditions remain insufficiently explored.

### 6.3. Nano-Enabled Irrigation and Sensing Systems

Nano-sensor technologies provide advanced tools for monitoring soil moisture and plant stress, enabling more precise and efficient irrigation management. Nano-enhanced sensors offer high sensitivity and spatial resolution, supporting real-time decision-making and improved water-use efficiency [132]. In addition to soil moisture monitoring, nano-sensors can detect early physiological indicators of drought stress, such as changes in plant metabolites and sap composition, allowing for timely intervention before visible symptoms occur. Integration of these sensors with automated irrigation systems can further optimize water application. However, practical implementation remains limited due to challenges related to sensor calibration, durability, maintenance, and integration with existing agricultural infrastructure [140]. Large-scale adoption therefore requires further technological refinement and cost reduction. Despite the rapidly expanding body of literature reporting the benefits of nanotechnology for drought mitigation, the robustness and real-world applicability of these findings remain a critical concern. A substantial proportion of reported improvements in plant drought tolerance, water-use efficiency, and soil-related functions are derived from controlled laboratory or greenhouse experiments conducted under simplified and highly regulated conditions. For instance, Kah et al. [145] and Lowry et al. [146] highlighted that many nano-enabled agricultural studies rely on short-term assays that do not adequately capture environmental variability, soil heterogeneity, or long-term ecological interactions. Moreover, Zhai et al. [147] emphasized that nanoparticle behavior, stability, and bioavailability can vary significantly under field conditions due to fluctuations in pH, salinity, and organic matter content. These factors collectively limit the direct translation of laboratory-scale efficiencies into consistent field performance, highlighting the urgent need for long-term, multi-location validation studies under realistic agricultural conditions.

## 7. Nanotechnology for Water Conservation

Similar challenges are evident in nano-enabled water treatment and desalination systems, where high removal efficiencies and flux rates are often achieved under controlled experimental conditions using simplified or synthetic water matrices. Foundational work by Shannon et al. [148] and Qu et al. [149] demonstrated the potential of nanomaterials in water purification; however, translating these results to real-world applications remains constrained by operational complexity and environmental variability. In addition, several critical barriers limit large-scale implementation, including the high cost and energy demand associated with nanomaterial synthesis, challenges related to material stability and recyclability, and uncertainties surrounding nanotoxicity and environmental fate, as discussed by Keller et al. [150] and Gottschalk et al. [151]. Addressing these constraints requires standardized evaluation frameworks, long-term performance assessments, and integration with existing water management systems to bridge the persistent gap between laboratory innovation and practical deployment. Nanotechnology can support water-saving through three major pathways: (1) nano-enabled filtration and purification; (2) smart, sensor-driven irrigation systems; and (3) nano-biostimulants that increase plant water-use efficiency. Nanotechnology contributes to water conservation by improving purification efficiency, enabling low-energy desalination, supporting water reuse, and reducing water losses. This contribution could be achieved through the following water conservation mechanisms: (I) Increased usable water supply by increasing efficient pollutant removal, enhancing selectivity and then increasing safe water supply. Nanomaterials such as carbon nanotubes, graphene, and metal oxides show high adsorption capacity, reactivity, and selectivity, enabling the removal of heavy metals, dyes, pharmaceuticals, pathogens, and nutrients [152]. (II) Nanomaterials have a high ability to enhance wastewater recycling through advanced filtration systems [20]. Expanded freshwater can be produced from desalination using nano-fluids, nano-filtration through nano-composite layers and 2D materials like graphene and hydrogels. These innovations lower energy demands, reduce salt fouling, and support off-grid desalination—especially valuable in water-scarce regions [153]. (III) Reducing water consumption reuse through rapid pollutant degradation, disinfection, catalytic nanomaterials using Zwitter-ionic hydrogels, TiO_2_ systems, and magnetic-NPs [154,155]. (IV) Lower operational water losses through fouling-resistant, durable membranes by using 2D MXene-polymer membrane nano-composites [156,157,158]. (V) Sustainable, decentralized purification by low-cost solar evaporation and nano-composite evaporators using graphene oxide-layered molybdenum boride (rGO-MBene) [159].

### 7.1. Nanomaterials for Water Purification

Emerging NMs can provide advanced solutions for water purification because of their large surface area, tunable surface chemistry, and strong catalytic and adsorption performance [160,161]. However, practical deployment remains constrained by challenges related to cost, scalability, durability, material recovery, and potential environmental risks. So, green synthesis, AI-assisted material design and lifecycle assessment are suggested approaches for improving sustainability and accelerating the translation to real-world applications [162]. The results of nano-enabled water purification technologies are distinguished and high-performance under laboratory conditions compared with real-world feasibility. The reasons may relate to using synthetic or ideal wastewater conditions; operating under controlled pH, temperature, and ionic strength; producing milligram-scale batches of NMs with precise structure and purity; and avoiding harsh cleaning regimes and multi-month continuous operation. This may explain why nano-membranes, nano-adsorbents, and nano-photocatalysts regularly achieve ultrafast flux >90–98% pollutant removal during laboratory trials. The main barriers preventing large-scale implementation might involve fabrication cost and economic barriers, long-term stability issues, manufacturing reproducibility and scale-up challenges. The common advanced NMs with strong lab performance but limited real-world feasibility are MXenes (2D carbides/nitrides), graphene and graphene oxide, metal–organic frameworks (MOFs), plasmonic NMs (Au, Ag), hybrid 2D/polymer nano-composite membranes, and biopolymer nano-composites and hydrogels [163,164].

### 7.2. Nanomaterials for Water Desalination

Nanotechnology plays a key role in enhancing water desalination processes through nano-structured membranes. Nano-structured membranes based on graphene, carbon nanotubes (CNTs), and MXenes have demonstrated superior permeability and selectivity compared to conventional membranes. These improvements are attributed to mechanisms such as size exclusion, controlled interlayer spacing, and Donnan exclusion, which enhance ion separation efficiency [165]. Surface functionalization further improves fouling resistance by reducing foulant adhesion and internal concentration polarization [160]. In addition, thin-film nano-composite membranes incorporating MXene interlayers have shown promising performance in groundwater desalination applications [166]. Despite these advances, several challenges limit practical implementation. These include fabrication complexity, material instability, high production costs, and a lack of standardized manufacturing protocols [161]. Furthermore, long-term environmental impacts and the recyclability of nanomaterials remain insufficiently understood. The major classes of NMs for water desalination that are used to enhance membrane and solar desalination systems might include carbon-based NMs (CNTs, graphene, activated carbon, carbon nano-fibers), metal and metal-oxide NMs, zeolites and nano-porous ceramics, electrospun nano-fibers and polymer nano-composites. The main advantages of laboratory-scale performance are higher water flux up to 100–110 L m^−2^·h^−1^ at high salt rejection, improved salt rejection, enhanced antifouling and antimicrobial behavior, high solar evaporation rates, and energy reduction. Many practical barriers limit large-scale implementation, such as high fabrication costs, long-term operational stability, reproducibility and manufacturing challenges, fouling and chlorine-induced degradation, and environmental and human-health risks [167,168].

### 7.3. Nano-Enabled Strategies in Water Management

Recent research has explored the integration of nanotechnology with alternative desalination and water treatment systems, including solar-driven desalination, capacitive deionization, and hybrid membrane processes. These approaches offer potential for energy-efficient and decentralized water treatment solutions [165]. Nano-enabled membranes can be incorporated into hybrid systems to enhance process efficiency and reduce energy consumption. However, most studies remain at the laboratory scale, and there is limited evidence on full-scale implementation and infrastructure integration [161,165]. In addition, integration with nature-based solutions and smart water management systems remains largely unexplored, highlighting a critical gap between technological potential and real-world application.

Two questions should be answered to explain the nano-enabled strategies: what nano-enabled strategies address drought mitigation, water conservation, and water/wastewater treatment, and how can these strategies be systematically classified, based strictly on the supplied literature? Multi-tiered classification of nano-enabled approaches for water-saving can be discussed based on their applications, mechanisms, and material platforms. Because no single source provides a unified taxonomy, this synthesis constructs an evidence-grounded classification from the explicit categories and technologies described across the retrieved documents. The first classification of nano-enabled strategies: these NMs could be classified into NMs for agricultural drought mitigation strategies for enhancing drought tolerance (like nano-priming), NMs for soil water conservation (e.g., nano-hydrogels, nano-biochar, and NPs-enhanced microbial biofertilizer), NMs for water treatment and purification to enable advanced treatment (nano-bioremediation, nano-filtration, and nano-membranes), and NMs for integrated water management and circularity (like nano-adsorbents) [169,170,171].

The second classification is based on the approaches for water-saving to be included photo-thermal/solar-driven interfacial evaporation technologies, such as enabling low-energy desalination and freshwater production (carbon-based photothermal absorbers, polymer–NP hybrids); nano-enabled membrane to modify or construct membranes to increase permeability, selectivity, antifouling, or stability, enabling water-saving through more efficient treatment or reuse (e.g., nano-porous membranes and nano-reinforced polymeric membranes incorporating CNTs); nano-enabled adsorption and sorption for efficient pollutant removal, reducing treatment time, chemical use, and water losses (e.g., nano-adsorbents, bio-nano hybrids combining biochar/biopolymers); nano-catalytic advanced oxidation processes to accelerate pollutant degradation (e.g., MOF-based catalysts, MOF-derived carbons, nano-confined catalytic membranes); nano-enhanced atmospheric water harvesting to capture water vapor from air with high efficiency (e.g., super-hygroscopic nano-hydrogels); nano-enabled demulsification and oily wastewater treatment to enhance the separation of oil–water emulsions, improving industrial water recycling (e.g., nano-adsorbents and nano-catalysts, magnetic nano-adsorbents); hybrid and intelligent nano-systems to maximize water-saving (e.g., nano-biohybrid systems for treatment, AI- and nano-enabled intelligent water-saving systems) [172,173,174].

### 7.4. Economic Feasibility of Nano-Applications

The cost and scalability of NMs for water-saving technologies are important, along with energy demand and maintenance requirements of NM-based water-saving/desalination systems and benchmarking them against conventional technologies. The economic feasibility of this nexus depends mainly on high initial investment, high synthesis costs, maintenance costs, potential cost reduction and economic uncertainty. The main factors limiting the costs and scalability of NMs for water-saving technologies are that many NMs require expensive precursors, energy-intensive synthesis, or multistep fabrication (CNTs, GO, MOFs and metal NPs often cost USD 10,000–100,000 per ton). Energy consumption accounts for up to 91% of total production cost in some NM classes. Reductions in performance are expected when moving from ideal batch lab conditions to real wastewater conditions due to the complex mixtures and ionic strength. On the other hand, green and waste-derived NMs can improve the cost profiles, as waste-derived precursors or agricultural residues can reduce costs and align with circular economy principles, lower costs and improved scalability [152,175,176,177]. The cost and scalability assessment is summarized in the following Table 2.

### 7.5. Nanotoxicity and Safety of Nanomaterials

The nanotoxicity and safety of NMs (e.g., carbon-based and metal-based NMs) for water-saving applications are a crucial issue, as they can pose different environmental risks, and regulatory frameworks shape their deployment in water-saving and water treatment systems. Carbon NMs may leach into treated water or concentrate in brines in desalination membranes, hydrogels, and evaporators. Their mobility and persistence mean they require strong containment and recovery strategies as they have risk profiles driven mainly by persistence, mobility, and structure-dependent toxicity. Carbon NMs can accumulate in aquatic systems, with concerns about bioaccumulation and toxicity to fish, microbes, and other aquatic species. So, C-NM regulatory assessments highlight the need for lifecycle analysis, mobility reduction via surface functionalization, and monitoring of discharge and persistence. Metal-based NMs (e.g., Ag, ZnO, TiO_2_, Fe/Mn oxides) used in water treatment and water-saving systems (e.g., solar desalination, fouling-resistant membranes, antimicrobial coatings) pose risks primarily via ion dissolution and reactive oxygen species (ROS). Metal-oxide NMs exhibit ROS-mediated toxicity, iono-regulatory stress, and developmental effects in fish and crustaceans. Metal-based NMs also show higher acute ecotoxicity and must therefore be carefully monitored for release or leaching during desalination or filtration operations. The main differences between carbon-based and metal-based NMs may be that carbon-based NMs cause oxidative stress and persistent, mobile, and multi-trophic toxicity, disrupt microbial metabolism, and need mobility-limiting functionalization, whereas metal-based NMs have high acute toxicity via ion metal release, ROS generation, and impacts on algae, fish, and crustaceans, and transformation alters the toxicity. Regulatory frameworks affecting deployment in water-saving technologies mainly depend on regulations from the USA (EPA, FDA) and the EU (REACH, Drinking Water Directive), as well as global frameworks (OECD, WHO, Asia-Pacific). Major risks of NMs in water-saving applications could be identified by several recurring ecological, health, and operational risks, such as (1) the nanotoxicity and ecological harm of metal-based NPs, carbon-based NMs, nano-adsorbents, and nano-catalysts; (2) environmental release and fate uncertainty; (3) human exposure risks; and (4) operational and process risks [178,179,180].

## 8. Limitations, Regulatory Challenges, and Research Gaps

Despite the growing interest in nanotechnology for drought mitigation and water conservation, several critical limitations constrain its practical application. The current body of literature, as reflected in recent reviews and experimental studies, highlights substantial gaps related to environmental safety, scalability, real-world validation, and regulatory frameworks. One of the primary concerns associated with nanotechnology is the potential for nanotoxicity and bioaccumulation. Existing studies emphasize that engineered nanomaterials may pose risks to non-target organisms, soil biota, and aquatic systems, particularly due to their high reactivity and persistence [140]. Furthermore, uncertainties remain regarding their long-term environmental fate, transport, and transformation in soil–water systems. These unresolved issues complicate risk assessment and raise concerns about potential human exposure through food and water pathways. Another key limitation involves the stability and durability of nanomaterials under environmental conditions. Reviews consistently report uncertainties related to the long-term chemical and structural stability of nanomaterials, particularly under varying pH, salinity, and temperature conditions [161,165]. In addition, questions regarding recyclability, degradation pathways, and lifecycle impacts remain insufficiently addressed. These factors significantly affect techno-economic feasibility and sustainability assessments.

Although NMs demonstrate promising performance at the laboratory scale, their large-scale production and application remain challenging. Fabrication complexity, high costs of nanomaterial synthesis (e.g., graphene, MXenes), and a lack of standardized production protocols limit their commercial viability [161,165]. These economic barriers are particularly critical in agricultural systems, where cost-effectiveness is essential for adoption by farmers. A major limitation highlighted across the literature is the discrepancy between laboratory findings and field performance. Most reported benefits of nanotechnology—such as enhanced plant growth, improved water-use efficiency, and increased stress tolerance—are derived from controlled experiments [140,144]. However, there is a lack of long-term, multi-site field studies that account for environmental variability, soil heterogeneity, and climate interactions. As a result, the reproducibility and scalability of these technologies under real-world conditions remain uncertain. Future work should focus on long-term field trials, standardized ecotoxicological assessments, dose–response relationships, lifecycle and techno-economic analyses, and integration with sustainable and nature-based solutions. Table 3 summarizes a list of some case studies on the role of nanomaterials for the mitigation of drought stress.

For nano-enabled systems such as nano-sensors and advanced membranes, technical limitations related to durability, calibration, and system integration remain significant. For example, nano-sensor networks require robust calibration, maintenance, and compatibility with existing irrigation infrastructure, which are not yet fully developed [140]. Similarly, while nano-structured membranes show enhanced performance in controlled settings, their integration into full-scale water treatment systems remains limited. The regulatory landscape for nanotechnology applications in agriculture and water systems is still evolving. The current literature highlights the absence of standardized testing protocols, safety guidelines, and regulatory benchmarks necessary for large-scale deployment [165]. In addition, trans-disciplinary coordination between scientific, industrial, and policy sectors remains insufficient, hindering the development of clear governance frameworks. Another important limitation is the lack of empirical data on public acceptance and stakeholder perception of nanotechnology in water and agricultural applications. Existing studies provide limited evidence regarding how farmers, consumers, and policymakers perceive potential risks associated with nanomaterials. This gap may hinder adoption, particularly in regions where trust in emerging technologies is low. A key cross-cutting limitation identified throughout this review is the persistent gap between laboratory-scale performance and real-world applicability, which remains one of the most critical barriers to the adoption of nano-enabled solutions in water and agricultural systems

## 9. Research Priorities and Future Directions

The lab-to-field gap in nanotechnology application is a real problem, as a large body of evidence across the retrieved references shows that nanotechnology for saving water faces a persistent lab-to-field gap. This gap is driven by three clusters of barriers: regulatory hurdles, farmer adoption challenges, and nanomaterial stability and behavior in real environments. Concerning the regulatory hurdles, there are many examples that could be mentioned in this context, such as hydrogel-based water-saving systems, which face unclear regulatory pathways and barriers related to safety testing and registration due to the lack of standardized protocols for assessing stability, leaching, and reuse, which regulators require before field deployment; regulatory frameworks for nano-agri-inputs remain fragmented, causing delays and uncertainty in commercializing nano-enabled water-saving products. Regarding farmer adoption challenges, farmer adoption is restricted if nanomaterials are expensive or difficult to apply due to high cost and complexity, limiting the adoption of nano-enabled water-retention systems; the high production costs of NMs (e.g., CNTs, graphene oxide) used for water treatment and retention limit practical field use; and many nano-based soil amendments (e.g., polymer nano-composites for water retention) still require industrial-scale dispersion control and manufacturing capacity not available to most farmers. Farmers often lack understanding of the performance, safety, or application methods for nano-enabled systems, slowing real-world uptake. Environmental stability barriers are common due to NMs losing performance under realistic water and soil chemistry and the instability of NMs in real water/soil environments, directly limiting water-saving functionality (e.g., graphene oxide). Many water treatment NMs show aggregation, fouling, or loss of activity under variable pH, temperature, and ionic strength typical of agricultural water sources. Under field conditions, soils can alter the behavior of NPs, reducing water-saving efficacy as soil-water interactions cause NP aggregation, passivation, or mobility loss, leading to unpredictable water-retention performance in field conditions. The recovery of NMs from water systems remains unsolved as NMs can escape into water streams, posing ecological risks; recovery requires additional filtration steps during disinfection or soil water retention enhancement [190,191,192].

The role of nanotechnology in drought mitigation and water conservation was discussed by several studies with a focus on the possible research priorities and future directions. These span plant-focused nano-interventions, soil/water system innovations, and cross-cutting issues such as scalability, safety, and regulatory readiness. To address these limitations, future research should focus on enhancing plant drought tolerance mechanistically through quantifying the relative contributions of NP-driven mechanisms (ROS scavenging vs. osmoregulation vs. nutrient biofortification), and understanding crop- and genotype-specific NP responses, including hormetic effects and toxicity thresholds [58]. Next-generation nano-formulations should be validated at the field scale under diverse agro-climatic conditions, along with the development of multifunctional nano-formulations in water retention, nutrient delivery and bio-protection [19]. Nanotechnology for water conservation and management should be developed by scaling nano-enabled water treatment to cost-effective, energy-efficient systems and the development of biodegradable or safer-by-design nanoparticles to mitigate toxicity concerns [193]. Nanotechnology–microbiome synergy is needed via the integration of NPs with PGPR to improve nutrient uptake, proline/sugar accumulation, and antioxidant defense under drought, along with developing nano-formulated microbial inoculants for improved stability and colonization [48]. Soil health, water retention and conservation, as important issues, should be investigated by focusing on soil–nano interactions across soil types and climatic regions, and long-term ecological impacts on soil microbiota. Advanced sensing and early stress detection are crucial, using nano-enabled sensors for the real-time monitoring of soil moisture, plant stress signals, and water quality, as well as integration with AI-driven decision-making for drought management and non-invasive, field-deployable nano-sensors for early drought stress detection. More research priorities could be listed as follows:

1. Long-term, multi-site field trials to validate laboratory findings.
2. Standardized ecotoxicological and risk assessment frameworks.
3. Comprehensive lifecycle and environmental impact analyses.
4. Development of cost-effective and scalable production methods.
5. Integration of nanotechnology with nature-based and conventional solutions.
6. Strengthening regulatory frameworks and stakeholder engagement.

## 10. Conclusions

Under a changing world, the global water crisis is a serious challenge facing all nations sooner or later. This challenge could be exaggerated by climate change and global drought. The impacts of drought on the global water crisis were reported to have reached the agricultural and industrial sectors, along with ecosystem and socio-economic issues. Nature-based drought solutions are effective strategies for facing such a crisis, along with nanotechnology. NMs can support wastewater recycling through nano-adsorbents and nano-filtration membranes that remove agrochemical residues, heavy metals, and pathogens, thereby promoting circular water use in farming systems. Nano-mitigation for drought is an essential strategy that can be achieved by enhancing plant stress tolerance, improving soil water retention, delivering nutrients more efficiently under water deficit, and supporting soil–microbe–plant interactions that strengthen drought tolerance. Nanotechnology for water conservation is also considered a crucial issue that can be adapted mainly in both agricultural and industrial sectors. Further deep research is needed in this very vital area for more sustainable and nature-based solutions with a focus on long-term, multi-site field trials, standardized ecotoxicological and risk assessment frameworks, comprehensive lifecycle and environmental impact analyses, and the integration of nanotechnology with nature-based and conventional solutions.

## Figures and Tables

**Table 1:** Main nano-enabled drought mitigation strategies.

Nano-Mitigation Pathway	Suggested Mechanisms	Suggested Effects	Refs.
Nano-enhanced plant drought tolerance	ROS scavenging, osmotic adjustment, improved photosynthesis and nutrient uptake	Increased WUE up to 52% and relative water content; reduced oxidative damage and improved growth and yield	[123]
Nano-soil conditioners and hydrogels	Increased water retention, porous nano-structures, enhanced soil aggregation	Higher soil water retention (up to 150% compared to control), improved porosity, reduced drought-induced stress	[125]
Nano-based water purification and desalination	Photo-thermal conversion, nano-fluids, nano-porous membranes, MXenes and graphene	Higher freshwater yield, improved solar desalination efficiency, enhanced salt resistance	[126]
Nano-enabled water management system	Smart irrigation, nano-filtration, nano-sensors	More efficient water use, improved reclaimed-water quality	[20]
Nano-biochar and nano-clays	High porosity enhances water holding, reduces salinity, improves moisture in coarse soils	Nano-biochar boosts water retention and plant resilience; nano-clay increases soil WHC	[127,128]
Nano-fertilizers (NFs)	Controlled nutrient release and water retention through porous carriers	NFs retain moisture 60–72% longer than controls; can raise soil WHC up to 80%	[129]
Metal and silica nanoparticles	Activate antioxidant enzymes, maintain membrane stability, increase RWC, regulate photosynthesis	Si, Zn, Fe, Mg nanoparticles enhance drought tolerance via ROS suppression and gene expression	[130,131]
Smart nano-enabled irrigation	Nano-sensors monitor soil moisture; nano-carriers deliver water and nutrients on demand	Wireless nano-sensors and nano-fertigation improve water-use efficiency	[132]
Plant physiological nano-enhancers	Improve osmolyte accumulation, stomatal regulation, antioxidant activity	NPs up-regulate drought-responsive genes and improve water relations	[133,134]
Combined nano-fertilizer and hydrogel systems	Synergistic moisture retention and nutrient efficiency	Nano-DAP and hydrogels increased soil WHC to ~79% and improved plant productivity	[135]

**Table 2:** The cost and scalability assessment of nano-applications for water desalination.

Nano-Application	Feasibility	Cost Profile	Key Limitations
Nano-membranes (RO/NF)	Medium–high	High capex, lower OPEX	High material cost, scaling-up issues
Solar nano-evaporators	High (small scale)	Low OPEX, modest CAPEX	Nano-fluid instability at scale
Nano-confined AOPs	Medium	60–75% cheaper than conventional AOPs	Needs pilot-scale validation
Microbial desalination cells	Medium	Lower operational cost	High material costs
MXene/graphene membranes	Low–medium	Very high cost	Poor large-scale viability
Coal-/biomass-derived nanomaterials	High	Low cost, scalable	Lower performance ceiling

Abbreviations: RO = Reverse Osmosis; NF = Nanofiltration; AOPs = Advanced Oxidation Processes; OPEX = Operational Expenditure; CAPEX = Capital Expenditure.

**Table 3:** A survey on some recent publications focused on nano-mitigation for drought.

Plant Species	Nanomaterial Info	Drought Details	Main Mechanism	Refs.
Wheat (*Triticum aestivum* L.)	Bio-Si-NPs (30, 60, 90, and 120 ppm)	Irrigation regimes with 100 and 50% soil moisture content	Mitigates the physiological changes and up-regulation of stress genes	[139]
Tomato (*Solanum lycopersicum* L.)	Nano-biochar (1, 3 and 5% *w*/*w*)	Irrigation at 100 and 60% field capacity	Improved biochemical attributes by 1% nano-biochar	[181]
Mulberry (*Morus alba* L.)	ZnO-NPs at 5, 10 and 50 mg/kg soil	Irrigation regime: every 2, 4, 6, 8 and 10 days	Induced growth by enzymatic and non-enzymatic antioxidants	[182]
Rose Carmine (*Echinacea purpurea* L.)	Foliar Se-NPs at four doses (0, 5, 10, and 20 mg L^−1^)	Drought stress: at four levels (20, 40, 60, and 100% of field capacity)	Enhanced morpho-physiological attributes and gene expression related to the phenyl–propanoid pathway	[183]
Coriander (*Coriandrum sativum* L.)	ZnO-NPs at 50 and 100 mg kg^−1^ primed with proline betaine	Control (unstressed) and irrigation upon wilting (stressed plants)	Accumulation of various phyto-chemicals and quenching of oxidative stress in plants under stress	[184]
Cluster bean (*Cyamopsis tetragonoloba* L.)	Foliar nano-K, Zn and B (2470, 1235, and 930 g ha^−1^)	Normal and skipped irrigation (starting from 41 days after sowing)	Nano-fertilizers improved growth, physiology, and yield and enhanced nutrient uptake	[129]
Cotton (*Gossypium hirsutum* L.)	Foliar ZnO-NPs at 25, 50, 100 and 200 mg L^−1^	Water regime: 75 and 50% soil relative water content	ZnO-NPs preserve chloroplast integrity and improve cotton leaf hydraulic traits	[185]
Tea (*Camellia sinensis* (L.) O. Kuntze)	Applied foliar of 0.1 mM nano-selenium (8 ppm)	Well-watered at 80% FC; drought stress at 35% field capacity (FC)	Enhanced N-translocation uptake rates; increased amino acids, soluble sugars and polyphenols	[186]
Rice (*Oryza sativa* L.)	Nano-biochar (1.0% *w*/*w*)	Drought stress at 20% of PEG 6000	Improving physiological and biochemical traits	[187]
Soybean (*Glycine max* L.)	Foliar ZnO-NPs at 50, 100, 200, and 400 mg L^−1^	Drought stress: nutrient solution containing 10% (*w*/*v*) PEG-6000	Low-dose ZnO-NPs (<200 ppm) enhanced photosynthetic efficiency (49.1%), while higher doses (>200 ppm) reduced it by 66.3%	[188]
Rapeseed (*Brassica napus* L.)	Foliar Ca-NPs at 100 mg L^−1^	Drought stress: nutrient solution containing 15% (*w*/*v*) PEG-6000	Ca-NPs up-regulated proteins associated with carbon fixation and chlorophyll metabolism	[189]

Abbreviation: PEG: Polyethylene Glycol.

## Data Availability

No new data were created or analyzed in this study.
